# Weight Loss Improves Liver Dysfunction and Dipstick Proteinuria in Obesity: The Japan Specific Health Checkups Study

**DOI:** 10.31662/jmaj.2023-0008

**Published:** 2023-06-30

**Authors:** Kei Nagai, Takuya Harada, Kaori Mase, Kunitoshi Iseki, Toshiki Moriyama, Kazuhiko Tsuruya, Shouichi Fujimoto, Ichiei Narita, Tsuneo Konta, Masahide Kondo, Masato Kasahara, Yugo Shibagaki, Koichi Asahi, Tsuyoshi Watanabe, Kunihiro Yamagata

**Affiliations:** 1 Department of Nephrology, Faculty of Medicine, University of Tsukuba, Tsukuba, Japan; 2 The Japan Specific Health Checkups study (J-SHC study) Group, Fukushima, Japan

**Keywords:** liver disease, chronic kidney disease, body mass index, obesity, exercise

## Abstract

**Introduction:**

Obesity and inappropriate lifestyle is the major risk factors for liver dysfunction and proteinuria. Nevertheless, previous studies have not described the differential impacts of body weight changes and lifestyle modification on already developed liver dysfunction and proteinuria.

**Methods:**

The original cohort was 933,490 individuals from the Japanese general population. In this investigation, we included 36,256 obese individuals with elevated levels of aspartate aminotransferase and/or alanine aminotransferase (≥31 IU/L) or positive proteinuria (+/− or more) in both the first and second years. Outcomes were the first normalization of these data defined as improvement in liver dysfunction and proteinuria. Times to outcomes were assessed using the Cox proportional hazards modeling for −1 kg/m^2^/year change in body mass index (BMI) changes in exercise and alcohol intake.

**Results:**

The multivariable-adjusted hazard ratio (HR) for incident improvement in liver dysfunction with BMI change −1.0 kg/m^2^/year was 1.07 (95% confidence interval [CI] 1.05-1.09) in obesity and that with improved proteinuria was 1.04 (95%CI 1.02-1.07). Compared to subjects without exercise habits, subjects who gained exercise habits exhibited a higher rate of improvement in liver dysfunction (HR 1.08; 95%CI 1.01-1.15) but not in proteinuria (HR 0.98; 95%CI 0.88-1.08). Compared to subjects with continuous alcohol intake habits, subjects who quit alcohol intake also showed a higher rate of improvement in liver dysfunction (HR 1.20; 95%CI 1.09-1.32).

**Conclusions:**

This study suggested that weight loss greater than 1 kg/m^2^/year improves liver dysfunction and dipstick proteinuria in obesity. Particularly, liver dysfunction can be remedied by acquiring an exercise habit and quitting alcohol intake.

## Introduction

Obesity is considered to increase the risk of developing major risk factors for liver dysfunction due to several liver disorders, including fatty liver ^(1)^. Obesity is also involved in the development of chronic kidney disease (CKD) under pathologies, such as diabetes mellitus and hypertension ^(2)^. The presence of both liver dysfunction and proteinuria as a phenotype of CKD is associated with metabolic syndrome ^(3), (4)^. Recently, focus has been placed on extrahepatic diseases in patients with fatty liver, one of which is CKD ^(5)^. Metabolic dysfunction-associated fatty liver disease (MAFLD) is strongly and independently associated with a 1.34-fold risk of prevalent CKD and abnormal albuminuria ^(3)^. Awareness of the risk of obesity and education promoting a healthy lifestyle, including proper nutrition and exercise, may help prevent fatty liver disease and the onset of CKD ^(2), (6)^.

Weight loss is important to combat the disease burdens associated with metabolic syndrome, which has been increasing globally. Lifestyle modification to achieve weight loss is advocated as definitively beneficial for all patients with fatty liver disease ^(7)^. Liver dysfunction is considered to be alleviated after interventional body weight loss ^(8), (9), (10)^ and adoption of increased physical activity ^(10), (11), (12)^. Similarly, proteinuria is significantly decreased in obese CKD patients following proper interventional weight loss ^(13), (14)^. However, previous studies have not described the differential impacts of body weight changes and lifestyle modification on already developed liver dysfunction and proteinuria.

The Japan Specific Health Checkups study group has recently reported that weight loss may decrease the incidence of proteinuria ^(15)^. Few reports appear to have clarified the effects of changes in body weight and exercise habits on the development of liver dysfunction and proteinuria in an obese cohort of the Japanese general population. We hypothesized that improvements in lifestyle, particularly in terms of reducing body weight, acquiring exercise habits, and quitting alcohol intake, would improve liver dysfunction and proteinuria among obese patients. To test this hypothesis, we investigated a large observational cohort of subjects undergoing specific health checkups in Japan.

## Materials and Methods

**Figure 1.**
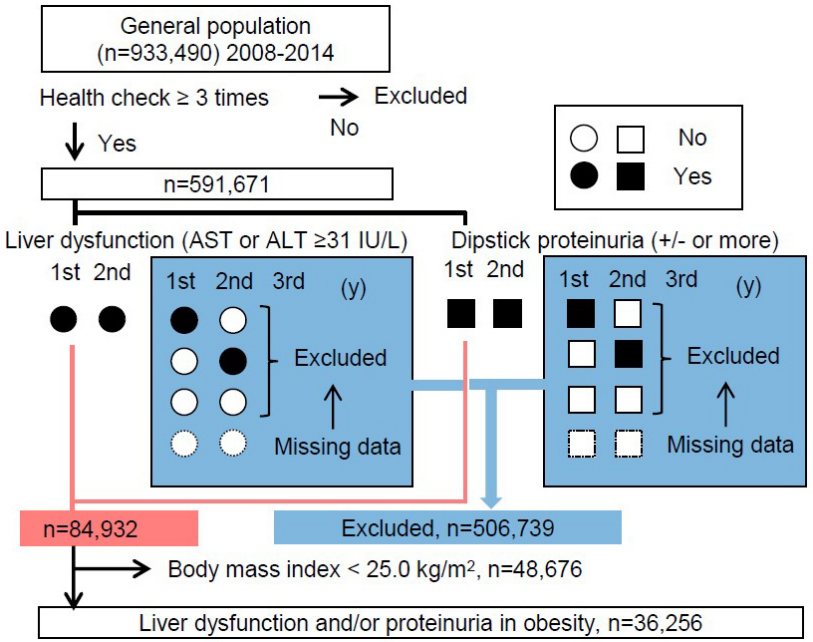
Inclusion and exclusion flow The cohort study originated with 933,490 individuals from the general population who had participated in annual specific health checkups since 2008. Subjects in the first analysis were thus 591,671 individuals (58.8% women) for whom all of the data necessary for this study were available. Since the purpose of this study was to determine whether weight loss is associated with improvements in liver dysfunction and proteinuria, analysis was further limited to 36,256 obese individuals (body mass index ≥ 25 kg/m^2^ at baseline) with elevated levels of aspartate aminotransferase (AST) and/or alanine aminotransferase (ALT) (≥31 IU/L each) or positive proteinuria (+/− or more) in both the first and second years of the study. Of these final subjects, 27,615 had persistent liver dysfunction, 11,396 had persistent proteinuria, and 2,755 were duplicates. Outcomes for analysis were improvement of liver dysfunction and/or proteinuria during follow-up with a mean interval of 3.02 years from the second survey.

The original study cohort was based on 933,490 individuals from the general Japanese population who had participated in annual specific health checkups since 2008 according to “The Specific Health Check and Guidance in Japan.” Most study participants were therefore relatively healthy, community-dwelling residents aged between 40 and 74 years. Since this investigation required sequential information to determine changes in body mass index (BMI), liver function, and urinary dipstick protein, participants with results available from less than three examinations were omitted from analyses. Subjects for the first analysis thus comprised 591,671 individuals (58.8% women) for whom all data necessary for this study were available, that is, information regarding age and sex; consecutive results for BMI, systolic blood pressure, diastolic blood pressure, habitual smoking and alcohol intake, and uses of antihypertensive drugs, lipid-lowering drugs, and hypoglycemic drugs; and relevant laboratory data. Since the purpose of this study was to determine whether weight loss is associated with improvements in liver dysfunction and proteinuria, the analysis was further limited to the 36,256 obese individuals showing elevated levels of aspartate aminotransferase (AST) and/or alanine aminotransferase (ALT) (≥31IU/L each) or positive proteinuria in both the first and second years of the study, and we designated them as persistent liver dysfunction or persistent proteinuria (Figure 1). Of these final subjects, 27,615 had persistent liver dysfunction, 11,396 had persistent proteinuria, and 2,755 were duplicates. The database was used and managed solely by the statistician, and data from the study cohort were obtained only after concluding memoranda with the municipal heads. All data were anonymized. Information transfers were coordinated through local government officials, and the standard analytical file (SAF) version 4.0 was developed based on the approved study protocol. The original ethics approval was obtained from Fukushima Medical University (approval nos. #1485 and #2771) and the institutional review board for ethical issues at the University of Tsukuba (approval no. 999; UMIN: 000019774). Further analyses were then performed using the SAF without any personal identifiers.

### Measurement of parameters and evaluation of comorbidities

Urinalysis by the dipstick method was performed on a single-spot urine specimen. Urine dipstick results were interpreted by the medical staff at each local medical institution and recorded as (−), (+/−), (+), (2+), or (3+). In Japan, the Japanese Committee for Clinical Laboratory Standards (http://jccls.org/) has proposed that all urine dipstick results of (+/−) should correspond to a urinary protein level of 15 mg/dL. In this study, proteinuria was thus defined as a result of (+/−) or more to prioritize detection sensitivity. Blood samples were assayed within 24 h using an automatic clinical chemical analyzer after collection. Using the enzymatic method, serum creatinine was measured. Annual change in BMI (ΔBMI) was determined using data obtained from measurements in the first and second years and divided by the interval between measurements in years (ΔBMI per year).

### Statistical analysis

The criteria for determining obesity differ from country to country, and the World Health Organization standard defines “obese” as a BMI of ≥30 kg/m^2^ ^(16)^. According to the standards set by the Japan Society for the Study of Obesity, we defined “obesity” in this study as a BMI of ≥25 kg/m^2^ ^(17)^. Based on our previous study, a reduction in BMI was defined as a change exceeding −1.0 kg/m^2^/year ^(15)^. The Specific Health Check includes a question regarding exercise habits using a binary question item: “Light sweaty exercise for at least 30 minutes at least 2 days a week for at least 1 year.” We utilized baseline and subsequent information to define “loss of habit” as a change from “yes” to “no” in each participant’s answers and “gain of habit” as a change from “no” to “yes.” Follow-up health checks were conducted through April 2015, as previously reported ^(18)^. Categorical variables are presented as numbers or percentages and continuous variables are presented as means and standard deviations (Table 1). Outcomes for analysis were the first normalization of liver dysfunction and/or proteinuria during follow-up (mean interval, 3.02 years from the second survey) among subpopulations divided by annual ΔBMI between the first and second surveys, with a mean interval of 1.2 years (Figure 1). This normalization of laboratory data changes is designated as “improvement” in this study. Times to outcomes were assessed using the Cox proportional hazards modeling for −1 kg/m^2^/year change in BMI and changes in exercise and alcohol intake with adjustment for age and sex, and further variables as follows: baseline BMI, estimated glomerular filtration rate, current smoking, current daily alcohol intake habit, systolic blood pressure, diastolic blood pressure, use of antihypertensive drugs, use of hypoglycemic drugs, use of lipid-lowering drugs, hemoglobin A1c, triglycerides, high-density lipoprotein, and low-density lipoprotein. Values of *p* < 0.05 were considered significant. Statistical analyses and graphical presentations were performed using SPSS version 27.

**Table 1.** Study Population with Liver Dysfunction and/or Proteinuria at Baseline.

Liver dysfunction and/or proteinuria (+)		Obesity (−)	Obesity (+)	*P* value
Study size	(persons)	48,676	36,256	
Sex	(% female)	35.1	39.5	<0.001
Age	(years)	63 ± 8	61 ± 8	<0.001
Body mass index	(kg/m^2^)	22.1 ± 2.1	27.9 ± 2.6	<0.001
Systolic blood pressure	(mmHg)	131 ± 18	136 ± 17	<0.001
Diastolic blood pressure	(mmHg)	78 ± 11	81± 11	<0.001
Aspartate aminotransferase	(IU/L)	35 ± 21	35 ± 18	0.004
Alanine aminotransferase	(IU/L)	34 ± 23	43 ± 25	<0.001
Baseline proteinuria, +/- or more	(% yes)	31.0	39.9	<0.001
Estimated GFR	(mL/min/1.73 m^2^)	75.4 ± 17.4	74.4 ± 17.2	<0.001
Free blood sugar	(mg/dL)	101 ± 25	108 ± 28	<0.001
Hemoglobin A1c	(%)	5.4 ± 0.8	5.7 ± 0.9	<0.001
Triglycerides	(mg/dL)	139 ± 113	168 ± 116	<0.001
High-density lipoprotein	(mg/dL)	62.3 ± 17.9	53.6 ± 13.3	<0.001
Low-density lipoprotein	(mg/dL)	120 ± 34	129 ± 32	<0.001
Exercise habit	(% yes)	36.4	42.0	<0.001
Smoking	(%)	22.2	21.1	<0.001
Alcohol intake habit, daily	(% yes)	35.4	36.4	0.003
Use of antihypertensive drugs	(%)	32.8	33.2	0.220
Use of hypoglycemic drugs	(%)	7.7	7.2	0.006
Use of lipid-lowering drugs	(%)	15.8	16.3	0.049
ΔBMI	(kg/m^2^/year)	+0.1 ± 0.8	-0.1 ± 1.0	<0.001
Time to endpoint	(days)	1,088 ± 545	1,104 ± 549	<0.001

**Abbreviations**: BMI, body mass index; GFR, glomerular filtration rate.

## Results

**Figure 2.**
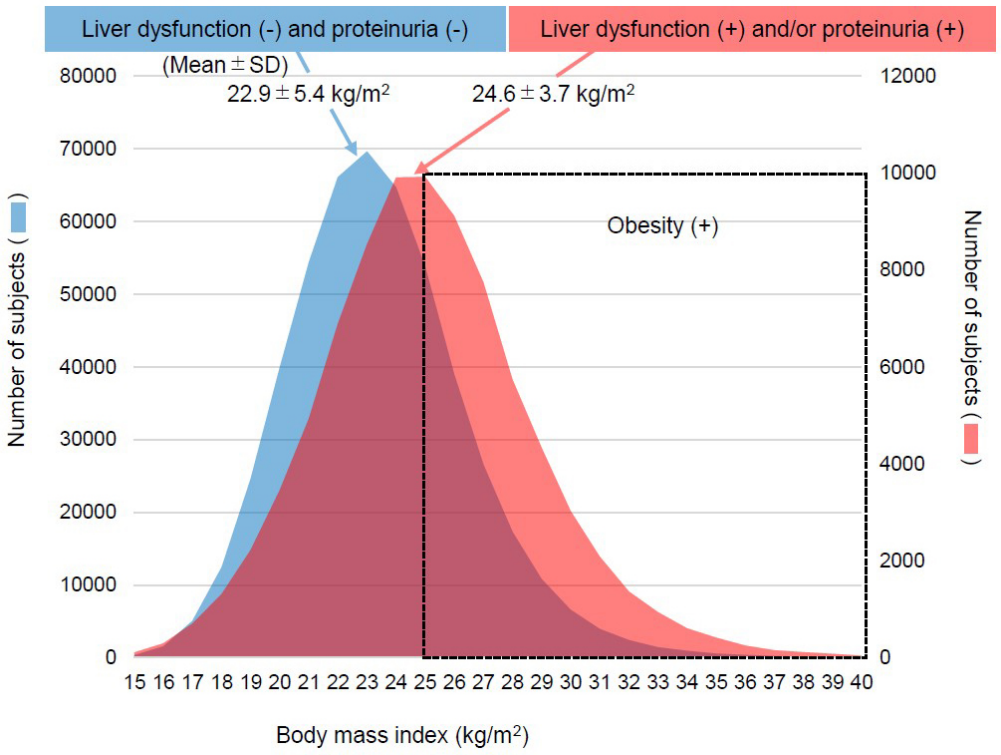
Distribution of body mass index with and without liver dysfunction and proteinuria Among the 591,671 subjects just after the first exclusion based on the number of health checks, we examined the distribution and mean body mass index at baseline for 506,739 subjects to be excluded based on the absence of both liver dysfunction and proteinuria and for 84,932 subjects with liver dysfunction or proteinuria, respectively. Obesity is defined as body mass index ≥ 25 kg/m^2^ at baseline.

Among the total of 933,490 individuals who underwent specific health checkups, 591,671 individuals who participated in more than three checkups were identified (Figure 1). Next, we examined BMI at baseline for the 506,739 subjects to be excluded based on an absence of both liver dysfunction and proteinuria and for the 84,932 subjects with liver dysfunction or proteinuria. The mean BMI of subjects with liver dysfunction or proteinuria (24.6 ± 3.7 kg/m^2^) was much higher than that in patients with the absence of these conditions (22.9 ± 5.4 kg/m^2^, *p* < 0.001) (Figure 2). Finally, our analysis was further limited to the 36,256 obese cases with persistent liver dysfunction and/or persistent proteinuria. The included obese cases presented younger age than the excluded 48,676 non-obese patients, and worse profile for high ALT, hypertension, dyslipidemia, and glucose intolerance (Table 1). The improvement was achieved in 13,199 out of 27,615 subjects who had persistent liver dysfunction and was done in 5,329 out of 11,396 subjects who had persistent proteinuria during follow-up. Subjects who did not achieve the outcome were censored at the final observation.

Furthermore, to determine the possibility of an independent effect of reducing BMI in the obese population, we investigated these results using age- and sex-adjusted and multivariable factor-adjusted Cox proportional hazards modeling (Table 2). The age- and sex-adjusted hazard ratio (HR) for improvement in liver dysfunction with −1.0 kg/m^2^/year of BMI reduction was 1.07 (95% confidence interval [CI] 1.05-1.09) in the obese population and HR for improvement in proteinuria was 1.04 (95%CI 1.02-1.07). Multivariable-adjusted HRs were also significant, at 1.07 (95%CI 1.05-1.09) for improvement in liver dysfunction and 1.04 (95%CI 1.02-1.07) for improvement in proteinuria.

**Table 2.** Age- and Sex-Adjusted and Multivariable-Adjusted Rates of Improvement in Liver Dysfunction and Proteinuria in Obesity.

		Improvement of liver dysfunction		Improvement of proteinuria	
Model 1	Units	Age- and sex-adjusted	*P* value	Age- and sex-adjusted	*P* value
Sex	female	1.07 (1.03-1.11)	<0.01	1.33 (1.26-1.40)	<0.01
Age	per −10 years	0.89 (0.87-0.91)	<0.01	1.09 (1.05-1.13)	<0.01
Δ Body mass index	per −1 kg/m^2^/year	1.07 (1.05-1.09)	<0.01	1.04 (1.02-1.07)	<0.01
Model 2		Multivariable-adjusted	*P* value	Multivariable-adjusted	*P* value
Sex	female	1.05 (1.00-1.10)	0.06	1.29 (1.20-1.40)	<0.01
Age	per −10 years	0.90 (0.87-0.93)	<0.01	1.00 (0.95-1.04)	0.89
Δ Body mass index	per −1 kg/m^2^/year	1.07 (1.05-1.09)	<0.01	1.04 (1.02-1.07)	<0.01
Body mass index	per −1 kg/m^2^	1.01 (1.00-1.02)	0.03	1.01 (1.00-1.02)	0.08
Estimated GFR	per +10 mL/min/1.73 m^2^	0.97 (0.96-0.99)	<0.01	1.07 (1.06-1.09)	<0.01
Smoking	no current smoking	1.01 (0.96-1.07)	0.59	1.08 (0.99-1.18)	0.07
Alcohol intake habit	not daily	1.01 (0.99-1.04)	0.32	1.01 (0.97-1.06)	0.55
Systolic blood pressure	per −10 mmHg	0.98 (0.96-1.00)	0.01	1.03 (1.01-1.05)	0.01
Diastolic blood pressure	per −10 mmHg	1.02 (0.99-1.04)	0.14	0.96 (0.93-1.00)	0.05
Use of antihypertensive drugs	no use	1.07 (1.03-1.12)	<0.01	1.27 (1.19-1.35)	<0.01
Use of hypoglycemic drugs	no use	0.98 (0.91-1.05)	0.54	1.17 (1.05-1.30)	<0.01
Use of lipid-lowering drugs	no use	0.99 (0.94-1.04)	0.69	1.10 (1.02-1.19)	0.02
Hemoglobin A1c	per −1%	0.96 (0.94-0.99)	0.01	1.09 (1.05-1.13)	<0.01
Triglycerides	per −10 mg/dL	1.00 (1.00-1.00)	<0.01	1.00 (1.00-1.01)	0.01
High-density lipoprotein	per −10 mg/dL	0.99 (0.97-1.01)	0.20	1.00 (0.98-1.04)	0.42
Low-density lipoprotein	per −10 mg/dL	0.99 (0.98-0.99)	<0.01	1.01 (1.00-1.02)	0.22

Cox proportional hazards model for −1 kg/m^2^/year change in BMI with adjustment for age and sex (Model 1), and further variables included as indicated in the table (Model 2). Abbreviations: GFR, glomerular filtration rate.

**Figure 3.**
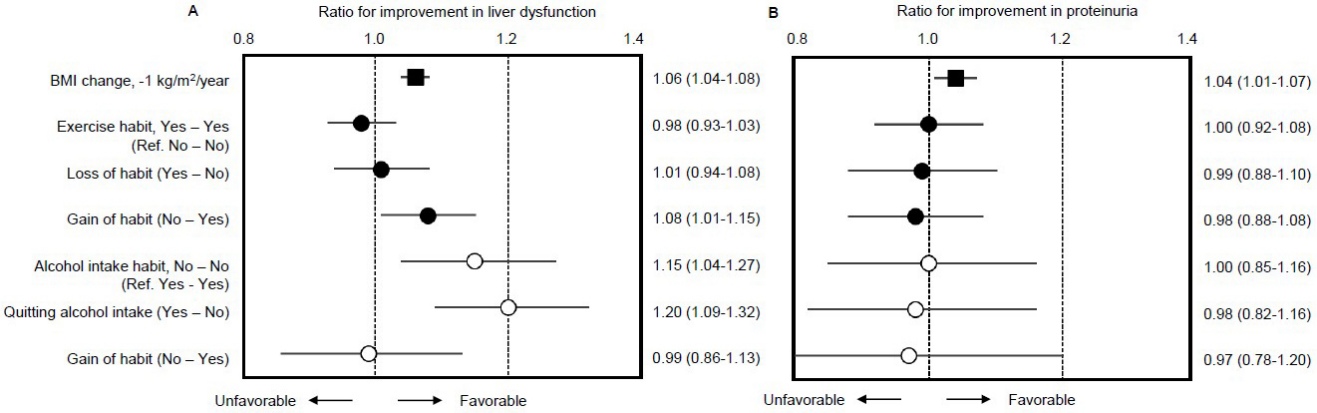
Multivariable-adjusted rates of improvement in liver dysfunction and proteinuria for the obese population with changes in body weight, exercise habit, and alcohol intake habit Outcomes for analysis were the first improvement of liver dysfunction (**A**) and proteinuria (**B**) during follow-up and times to outcomes were assessed by Cox proportional hazards modeling with adjustment for factors described in the **Methods** section and shown in Table 2. Multivariable-adjusted hazard ratios and 95% confidence intervals for improvement are presented. The Specific Health Check questionnaire includes questions regarding lifestyle and habits using question items: “Light sweaty exercise for at least 30 minutes at least 2 days a week for at least 1 year” and “Current alcohol intake status.” We utilized baseline and subsequent information to define “Loss of habit” as a change from “yes” to “no” in participant answers and “gain of habit” as a change from “no” to “yes,” respectively.

Finally, we examined the HRs of subjects with changes in exercise and alcohol intake as lifestyle factors using a multivariable-adjusted model (Figure 3A and 3B). Compared to subjects lacking exercise habits in both the first and second surveys (designated as “No-No”), subjects who gained an exercise habit (“No-Yes”) exhibited a higher rate of improvement in liver dysfunction (HR 1.08; 95%CI 1.01-1.15). Compared to subjects with continuous alcohol intake habits (“Yes-Yes”), subjects who quit alcohol intake (“Yes-No”) also showed a higher rate of improvement (HR 1.20; 95%CI 1.09-1.32) (Figure 3A). However, inconsistent with BMI change as a positive control for improvement in proteinuria (HR 1.04; 95%CI 1.01-1.07), neither gaining an exercise habit (HR 0.98; 95%CI 0.88-1.08) nor quitting alcohol intake (HR 0.98; 95%CI 0.82-1.16) impacted improvement in proteinuria within the mean 3.02 years of follow-up in this study (Figure 3B).

## Discussion

The worldwide prevalence of overweight and obesity is both high and increasing ^(19)^. Additionally, metabolic syndrome is commonly associated with both chronic liver disease and CKD presenting as consistent proteinuria ^(2), (20)^. Various phenotypes of liver and kidney diseases are associated with obesity and metabolic syndrome, including nonalcoholic fatty liver disease (NAFLD), nonalcoholic steatohepatitis, hepatocellular carcinoma, obesity-related glomerulopathy, urolithiasis, kidney cancer, and cardio-renal syndrome ^(2), (20)^. Among these, fatty liver disease is considered the predominant phenotype because 50%-80% of individuals with obesity also have NAFLD ^(21)^, compared to only 16% of individuals with normal BMI but lacking metabolic risk factors ^(22)^. Recently, the term MAFLD has been proposed to express the concept of liver diseases associated with known metabolic dysfunction ^(23), (24)^. AST and ALT levels were significantly higher in subjects who developed fatty liver disease than in those with non-fatty liver disease ^(25)^, and these levels thus provide significant biomarkers for the effect of weight loss interventions among individuals with NAFLD ^(6)^. Although obesity is not an essential precondition for proteinuria, weight gain concurrent with hypertension and glucose intolerance may be responsible for proteinuria as a proxy for kidney injury ^(26), (27)^. Based on this background, this study tested the impact of achieving weight loss and lifestyle modification on already developed metabolic-associated liver dysfunction (i.e., elevated levels of AST and/or ALT) and proteinuria. In our investigation, weight loss in the obese population appeared associated with better improvement in liver dysfunction (multivariable-adjusted HR 1.07, 95%CI 1.05-1.09 per −1 kg/m^2^/year), suggesting that metabolic-associated liver damage and kidney injury may be reversible among obese patients who are losing weight for whatever reason, interventional or non-interventional, consistent with previous studies in the context of NAFLD ^(6), (25)^.

Regarding the linkage between MAFLD and CKD, MAFLD offers a better identifier of patients with CKD than NAFLD, and MAFLD is strongly and independently associated with CKD and abnormal albuminuria ^(3)^. Moreover, one study demonstrated that individuals with fatty liver disease without metabolic syndrome were not at risk for the presence or incidence of CKD ^(5)^. This observation suggests the possibility of a shared mechanism between MAFLD and CKD, such as insulin resistance ^(28)^. Fatty liver diseases may exacerbate hepatic insulin resistance, promote hypertension, induce atherogenic dyslipidemia, and release a variety of pro-inflammatory molecules as pro-oxidant and pro-fibrogenic mediators that play important roles in the pathophysiology of CKD and other extrahepatic vascular complications ^(29), (30)^. A recent worldwide meta-analysis ^(30)^ identified only one study ^(31)^ examining the association between fatty liver associated with obesity and CKD in the Japanese population, so further research from Japan is sorely necessary.

In Japan, obesity is defined as a BMI of ≥25 kg/m^2^, and the presence of health problems requiring weight loss is also considered to be “obesity” ^(17)^. In Japan, few patients are severely obese with a BMI of >30 kg/m^2^, and although subjects receiving anti-obesity pharmacotherapy and weight-loss surgery are increasing, the numbers of individuals taking such steps remain relatively low and very few cases have been reported. Therefore, in reality, lifestyle interventions such as exercise and changes to dietary habits ^(32), (33)^ are likely to remain the mainstay of interventions for obese CKD patients in Japan for some time. Moreover, we clarified that the groups with a gain in exercise habits or quitting alcohol intake showed a significantly higher probability of improvement in liver dysfunction, but not improvements in proteinuria, than reference groups (Figure 3). Despite the fact that observational studies provide a lower level of evidence than randomized controlled trials, this result obtained from a large, general population cohort appears very useful in practice for obese populations or in providing health guidance after medical checkups in Japan. Although there is evidence that exercise does not increase urinary protein levels in CKD patients ^(34), (35)^, no studies have provided clear evidence that exercise reduces urinary protein. Similarly, concluding whether alcohol intake contributes to the amelioration of CKD presenting with urinary protein is also difficult based on current evidence. Instead, some evidence suggests that a small amount of alcohol in an individual with normal body weight (BMI 18.5-25 kg/m^2^) has suppressive effects on the development of CKD ^(36)^. Clear evidence is therefore currently lacking as to whether quitting alcohol intake is effective for reducing CKD and urinary protein. Nevertheless, this study had a mean observation period of only approximately 3 years, making it difficult to determine whether urinary protein is affected by the acquisition of exercise habits and quitting alcohol intake. We speculate that a longer observation period may reveal improvements in urinary protein with such lifestyle changes.

A key strength of this study was that to the best of our knowledge, it represents the first attempt to compare how body weight changes benefit Japanese individuals between those who have already developed liver dysfunction or proteinuria. However, some limitations must be kept in mind when interpreting the present findings. First, the implications of healthy weight loss as the main scope of the study, are very different when the fact of weight loss is due to complications and comorbidities. Second, since ultrasound results were not included in the specific health checkups, the diagnosis of fatty liver was only inferred from the coexistence of obesity and liver damage. Some research has pointed to liver dysfunction, obesity, and elevated ALT as significant predictors of NAFLD ^(37)^ and NAFLD is also diagnosed at a fairly high rate in liver disorders with obesity ^(22), (38)^. In this study, elevated liver enzymes in obese patients were considered as a surrogate marker for fatty liver. Third, γ-glutamyl transpeptidase is considered useful in the diagnosis of fatty liver as a marker of liver fibrosis but could not be included in this evaluation because of a lack of data during the follow-up period. Fourth, the relationship between weight change and eating habits was unclear due to a high frequency of missing data on eating habits, and clarification of the contribution of improved eating habits to weight change was not possible. Fifth, the obese population with liver dysfunction and/or proteinuria at baseline was so small that we were unable to elucidate whether weight changes and exercise habits independently affected improvement in liver dysfunction and proteinuria or instead affect these in relation to each other. Sixth, information regarding the use of drugs and lifestyle factors (i.e., smoking, exercise, and alcohol intake) were obtained via a self-reported questionnaire due to the design of the specific health checkups. Seventh, in the context of CKD, the incidence of adverse clinical outcomes differs significantly depending on the presence and severity of proteinuria. Because this is a cohort study utilizing a relatively healthy general population, the results are presented based on an analysis of persons with abnormal laboratory examinations regardless of the severity of proteinuria and renal dysfunction rather than primarily patients with advanced kidney disease who have a massive urinary protein. Eighth, since drug treatment for hypertension, hyperlipidemia, and diabetes may affect proteinuria, it would be interesting to track the number of years of treatment and the resulting changes in blood pressure, hyperlipidemia, and blood glucose after baseline, but this was not possible because of the design of the current study.

In conclusion, this observational cohort study suggested that weight loss equal to or greater than 1 kg/m^2^/year improves liver dysfunction and dipstick proteinuria in obesity. Particularly, liver dysfunction differs from urinary protein in that alleviation can be rapidly achieved with the acquisition of exercise habits and quitting alcohol intake.

## Article Information

### Conflicts of Interest

None

### Sources of Funding

This work was supported by Grants-in-Aid for “Research on Advanced Chronic Kidney Disease (REACH-J), Practical Research Project for Renal Disease” from the Japan Agency for Medical Research and Development (AMED) under grant numbers JP17ek0310005 and JP20ek0310010. This work was also supported by a Health and Labor Sciences Research Grant for “Study on the Design of the Comprehensive Health Care System for Chronic Kidney Disease (CKD) based on the individual risk assessment by Specific Health Check-Up” from the Ministry of Health, Labour and Welfare of Japan under grant number H24-nanchitou(jin)-ippan-006.

### Acknowledgement

This study would not have been possible without the generous support of the public health nurses and the officials in each district. The authors would also like to thank Ikuko Takano and Ayumi Kaichi for their secretarial assistance.

### Author Contributions

Conceived and designed the study: KN and KY. Analyzed the data: KN. Collected the data: KN, KY, KA, and TW. Wrote the first draft of the manuscript: KN. Contributed to the writing and editing of the manuscript: TH, KM, KI, TM, KT, SF, IN, TK, MK, MK, YS, KA, TW, and KY. All authors agree with the manuscript results, conclusions, and publication.

### IRB Approval and Consent

The original ethics approval was obtained from Fukushima Medical University (approval nos. #1485 and #2771) and the institutional review board for ethical issues at the University of Tsukuba (approval no. 999; UMIN: 000019774). According to this approval, an official memorandum of understanding was exchanged between the institutional head and each mayor of the local government that owns the health checkup information from citizens. The protocol waives the need for individual consent because all data were obtained after concluding memoranda with the municipal heads. Information transfer was coordinated through local government officials, and only the output form without any individual data was disclosed to researchers. The names, addresses, and all other personalized data of participants were completely deleted from the linked data to protect privacy.

### Availability of Data and Material

The data that support the findings of this study are available upon request to the last author (KY).
